# Monkeypox: context, implications and challenges for health services and surveillance

**DOI:** 10.1590/S2237-96222023000100018

**Published:** 2023-02-10

**Authors:** Laylla Ribeiro Macedo, Ethel Leonor Noia Maciel

**Affiliations:** 1 Universidade Federal do Espírito Santo, Laboratório de Epidemiologia, Vitória, ES, Brazil

In May 2022, a confirmed case of monkeypox in an individual with a history of travel to Nigeria, Africa, was reported in the United Kingdom. A few months after the first notification of the disease in Europe, the World Health Organization (WHO) Bulletin, published in early August, showed that 89 countries accounted for more than 27,000 cases and 11 deaths.^(1)^ With the progressive increase in new cases, on July 23, 2022, the WHO declared the disease a Public Health Emergency of International Concern (PHEIC), warning of the need to increase capacity to control the spread of monkeypox in the countries.^(2)^ In Brazil, on June 9, 2022, the first case of the disease was reported, and on August 29, 2022, the Ministry of Health announced that there were 4,693 confirmed cases in 24 Federative Units, in addition to one death in Minas Gerais.^(3)^

The monkeypox virus has been known since 1958, when it was discovered in Africa and named according to the criteria prevailing at the time. WHO recommends, as current best practice in naming diseases, that, for newly identified viruses and virus variants, should be given names with the aim to avoid causing offense to any cultural, social, professional or ethnic groups, regions or countries, in order to minimize any negative impacts on trade, travel or animal welfare. Therefore, the WHO has a process underway for the new name of the disease.^(5)^

In view of this issue, there was a debate amongst virologists and public health experts, during which they reviewed the terminology of monkeypox variants. After studying the evolutionary biology of the virus and the phylogenetic and clinical differences of the variants, they concluded that the proper naming structure would be represented by a Roman numeral for the clade, and a lower-case alphanumeric character for the subclades, with Clade one (I) referring to the former Congo Basin (Central African) clade, and Clade two (II), referring to the former West African clade, which consists of two subclades, Clade IIa and Clade IIb. Clade IIb refers to the group of variants that has been circulating in the 2022 global outbreak. However, these terms may be revised as new variants are eventually identified.^(5)^

Taking into consideration that monkeypox is not a new disease but a change in the disease pattern, the use of vaccines and medications for its prevention and treatment is present-day reality. The study that analyzed the efficacy of smallpox vaccine against monkeypox was published in 1988, using data on cases diagnosed between 1980 and 1984 in Zaire, Africa, and showed 85% protection against monkeypox for individuals.^(6)^ However, further studies are needed in order to investigate the protection of existing vaccines against the current Clade IIb. On August 25, 2022, the Brazilian Health Regulatory Agency (*Agência Nacional de Vigilância Sanitária* – ANVISA) approved the temporary waiver from registration for importation and use of monkeypox vaccine; however, at the time we finished this article, there was no medication available for the treatment of the disease on a large scale in the country.^(7)^

Although the number of cases has increased significantly in Brazil, monkeypox has not been declared a Public Health Emergency of National Concern (PHENC) yet. As regulated by Decree No. 7,616 of November 17, 2011, a disease can be announced as PHENC in situations that require the urgent use of measures to prevent and control risks, damages and injuries to public health. This measure aims to develop actions to combat emergencies such as monkeypox, and may accelerate and contribute to the establishment of agreements for the acquisition of vaccines and/ or medicines, as well as the expansion of the testing network, provided by the public health network, and its inclusion in the list of tests funded by health insurance plans.^(8)^

In the current scenario, eight laboratories in the country perform diagnostic tests for monkeypox detection, four of which are Central Public Health Laboratories (*Laboratório Central de Saúde Pública* – LACEN) and four other reference centers: two at Fundação Oswaldo Cruz (Fiocruz), one at the Universidade Federal do Rio de Janeiro (UFRJ) and one at Instituto Evandro Chagas (IEC). These laboratories are responsible for the national testing coverage, which causes slowness in the availability of test results and, consequently, several suspected cases without laboratory confirmation.^(8)^

In this context, it is extremely important to evaluate the demand for hiring human resources and services in an agile manner, mobilization of financial resources for training professionals and the relevant acquisition of inputs, as well as investment in information campaigns targeting the general population. In this sense, the declaration of monkeypox as PHENC represents an assertive and beneficial measure, in order to contribute to a timely response to the control of the disease in the country.^(8)^

The Ministry of Health, through the Public Health Emergency Operations Center (*Centro de Operações de Emergência em Saúde Pública* – COE Monkeypox), has developed a National Contingency Plan for monkeypox, whose main objective is to provide health professionals and managers with strategic information for containment, control and care, epidemiological and laboratory guidelines, useful for emergency management.^(10)^ The COE Monkeypox started operating on July 29, 2022, aimed at organizing the actions of the Brazilian National Health System (*Sistema Único de Saúde* – SUS) in a coordinated way, involving the three spheres of government in the response to the disease. The COE Monkeypox is comprised of representatives from the Ministry of Health, National Council of Health Secretaries (*Conselho Nacional de Secretarias de Saúde* – CONASS), National Council of Municipal Health Secretaries (*Conselho Nacional de Secretarias de Saúde* – CONASEMS), Fiocruz, ANVISA and Pan American Health Organization (PAHO).^(10)^However, it is recognized the need for members of the scientific community to integrate the COE.

It is noteworthy that structuring the Contingency Plan is as relevant as the wide dissemination of its content to health surveillance professionals and other services, in order to direct strategies to cope with the disease. It is also important the permanent review of this Plan by experts, aiming to monitor the speed of the changes experienced.

Timely access to data on monkeypox cases and deaths, so that they truly reflect their epidemiological context, has proved to be a challenge. The disease indicators in the country may be underestimated, due to testing barriers, lack of broad information campaigns about its symptoms, as well as referral guidelines to reference services and their care flows. Continuous knowledge and analysis of the indicators of incidence, mortality and hospital bed coverage, among others, are indispensable tools for action planning and goal setting by managers and other professionals involved. It is worth mentioning: it is essential that efforts should be made to ensure the reliability of these data, for a timely response based on the previously established plan; and that managers, professionals and civil society understand the characteristics of the disease and share the indicators obtained. The population plays an important role in this process, in the awareness and dissemination of information based on scientific evidence, as well as the adherence to measures that minimize exposure to the virus and the spread of the disease.^(8)^

It is also worth noting that the WHO recommends isolating individuals with monkeypox, cleaning surfaces or rooms frequently, separating utensils, washing bed sheets and bath towels in hot water, maintaining well-ventilated environments and regular handwashing, as the main measures to prevent the spread of infection. However, the implementation of these actions is complex when socioeconomic inequality in the country is observed. Thus, identifying these inequalities and targeting actions to groups characterized by any type of vulnerability, either by biological factors or by social differences, is another challenge for services.^(12)^

It is worth remembering that although monkeypox has been known since the late 1950s, the actions aimed at its control have been incipient over the years. It is a disease characterized by gross negligence and little investment of financial resources by the pharmaceutical industries, or even by large research funding agencies. In summary, the current panorama of monkeypox management could be more promising if effective preventive measures had been developed and implemented since its discovery in the African continent.

In conclusion, it is essential that the presence and health risks attributed to monkeypox are widely disseminated by the scientific community and institutions involved; and with equal effort, analyzed new pathogenic microorganisms, when identified in a certain locality/region, this is indeed a global public health issue, in view of its rapid dissemination potential to other regions of the world, given the huge susceptibility of integrated populations at the global level. This is a valuable lesson to be strongly considered in the current world health panorama, in order to avoid repeating the same mistakes in the future.

## References

[1] World Health Organization (2022). Multi-country outbreak of monkeypox, External situation report.

[2] Organização Pan-Americana da Saúde (2022). Diretor-geral da OMS declara que surto de monkeypox constitui uma
emergência de saúde pública de importância internacional.

[3] Ministério da Saúde (BR) (2022). COE Monkeypox. Card Situação Epidemiológica de Monkeypox no Brasil n° 42
SE 35.

[4] Menezes YR, Miranda AB (2022). Severe disseminated clinical presentation of monkeypox virus
infection in an immunosuppressed patient: first death report in
Brazil. Rev Soc Bras Med Trop.

[5] World Health Organization (2022). Monkeypox: experts gives virus variantnem names.

[6] Fine PE, Jezek Z, Grab B, Dixon H (1988). The Transmission Potential of Monkeypox Virus in Human
Populations. Int J Epidemiol.

[7] Ministério da Saúde (BR), Agência Nacional de Vigilância Sanitária (2022). Anvisa aprova liberação de vacina para monkeypox para uso pelo
Ministério da Saúde.

[8] Maciel E (2022). Espin é medida positiva e necessária para conter monkeypox.

[9] Tanure A (2022). Brasil faz 8.850 testes de varíola dos macacos; são 3.100 casos.

[10] Ministério da Saúde (BR), Plano de Contingência Nacional para Monkeypox (2022). Centro de Operações de Emergência em Saúde Pública: COE
Monkeypox.

[11] Maciel E (2022). Opinião. Monkeypox: Brasil segue falhando na testagem, no isolamento e
na vacinação.

[12] World Health Organization (2022). Monkeypox outbreak.

